# Supplementary material for: Effectiveness and trend forecasting of tuberculosis diagnosis after the introduction of GeneXpert in a city in south-eastern Brazil
Source: PLoS One. 2021 May 28;16(5):e0252375. doi: 10.1371/journal.pone.0252375 (PMC8162696; doi:10.1371/journal.pone.0252375)
Supplement: Attachment — Submitted filename: Comments-29-11-20.docx [file pone.0252375.s002.docx]

**Comments:**

1. English needs to be improved throughout the manuscript. There are few few instances, where the meaning is not clear. Ist line of Introduction, ‘is the main cause of death among people living with HIV (human immunodeficiency virus) (PLHIV), overcoming AIDS (Acquired Immunodeficiency Syndrome) as the most lethal infectious disease of today[1]’ needs to be changed.

2. Please change the ‘behavior of disease’ to better phrase..

3. We already have WHO TB report of 2020, please quote that instead of WHO (2019).

4.What do you understand by bacilloscopy? Do you mean smear microscopy? Smear microscopy is the most commonly used term. Pl use that.

5. Introduction, line 60, ‘slow reproduction of the bacillus’ is wrong. It is the turnaround time of *M. tuberculosis*, which is 4-8 weeks, though the generation time is ~ 18-20 hrs. Pl correct this.

6. Introduction, line 66, there is no need to define DNA. It is such a common term.

7. To the best I know, TRM-TB is not very common term in many parts of the world. I will avoid using this term. Please write this in full form.

8. Tuberculosis (TB) shall be abbreviated at the first time and then it shall be written as ‘TB’.

9. In Discussion, lines 257-259, ‘Studies show that for samples with negative smear microscopy, the sensitivity of the TRM-TB for a sputum sample is 72.5% and for three samples it reaches almost 91%[2]’

Please rewrite this sentence.

10. What is SUS? In line 268 same page. I believe, it is city. Then write accordingly.

11. Across the world, sputum smear microscopy and culture are commonly used methods for the detection of pulmonary TB. For the detection of extrapulmonary TB, these methods are not good. In fact, TB diagnosis also relies on other diagnostic modalities, like hisopathology, imaging, IGRAs including molecular tests, like LAMP test, PCR, multiplex PCR, real-time PCR, etc.

12. In Discussion, lines 274, ‘A culture test can detect between 70% and 90% of cases; however, it requires at least 14 to 60 days to obtain a diagnosis’ is not written accurate. In fact, the sensitivity of culture in extrapulonary paucibacillary TB specimens varies from 0-80%.

13. In Discussion, line 297, what do you mean by sensitivity test (ST)? Is it drug susceptibility test (DST)?

14. You need to compare Xpert assay at the Lab level with other routine tests like imaging, histopathology, cytology, PCR, real-time PCR, etc.Pl mention these tests in the Introduction as well as Discussion.
