# Supplementary material for: Effectiveness and trend forecasting of tuberculosis diagnosis after the introduction of GeneXpert in a city in south-eastern Brazil
Source: PLoS One. 2021 May 28;16(5):e0252375. doi: 10.1371/journal.pone.0252375 (PMC8162696; doi:10.1371/journal.pone.0252375)
Supplement: Attachment — Submitted filename: Response to Reviewers.docx [file pone.0252375.s003.docx]

**Dear Editor,**

Many thanks for your reply and your reviewers' comments about our manuscript **“PONE-D-20-34732 Effectiveness and trend forecasting of tuberculosis diagnosis after the introduction of GeneXpert® in a city in south-eastern Brazil”.**

The comments were appropriate to qualify/ improve the manuscript. We have forwarded a letter with the changes made in the manuscript, presenting our response for each comment from reviewers, as requested. Many thanks for the comments.

In the current version presented to the journal, we modified the question regarding data availability. Initially, they were collected at the request and approval of an ethics committee, but for the analyzes performed and presented in the present manuscript, we did not use any variable that could identify the cases included in the study. In this way, as the data is unrestricted, we upload the minimum set of anonymous data to replicate the results of the study, as requested.

Regarding the figures and tables of supporting information, there was a mistake on our part and we apologize, this has already been corrected. The figures and tables that we had placed in the supporting information section are actually the figures and tables present in the manuscript itself. There is only one supplementary document that is the minimum dataset, we have already corrected this error in the manuscript and in the system.

Below, we respond point by point to the comments of Reviewer #1 comments and would like to thank Reviewer #2 for spending time reading and reviewing our manuscript.

Kind regards,

Berra et al.

**Reviewers' comments:**

**Reviewer #1:**

1. **English needs to be improved throughout the manuscript. There are few instances, where the meaning is not clear. Ist line of Introduction, ‘is the main cause of death among people living with HIV (human immunodeficiency virus) (PLHIV), overcoming AIDS (Acquired Immunodeficiency Syndrome) as the most lethal infectious disease of today[1]’ needs to be changed.**

*Thank you for your comment. English was extensively revised throughout the manuscript and the first paragraph of the manuscript was reformulated to facilitate understanding.*

2. **Please change the ‘behavior of disease’ to better frase.**

*Thank you for your comment. This term has been changed throughout the manuscript.*

3. **We already have WHO TB report of 2020, please quote that instead of WHO (2019).**

*Thank you for your comment. The reference and information have been updated.*

4. **What do you understand by bacilloscopy? Do you mean smear microscopy? Smear microscopy is the most commonly used term. Pl use that.**

*Thank you for your comment. Bacilloscopy is the same as smear microscopy. The term "bacilloscopy" was changed throughout the manuscript and replaced with smear microscopy, as suggested.*

5. **Introduction, line 60, ‘slow reproduction of the bacillus’ is wrong. It is the turnaround time of M. tuberculosis, which is 4-8 weeks, though the generation time is ~ 18-20 hrs. Pl correct this.**

*Thank you for your comment. This has been fixed.*

6. **Introduction, line 66, there is no need to define DNA. It is such a common term.**

*Thank you for your comment. The definition of DNA has been removed as suggested.*

7. **To the best I know, TRM-TB is not very common term in many parts of the world. I will avoid using this term. Please write this in full form.**

*Thank you for your comment. Throughout the manuscript, the acronym TRM-TB was changed to rapid molecular test for TB.*

8. **Tuberculosis (TB) shall be abbreviated at the first time and then it shall be written as ‘TB’.**

*Thank you for your comment. This has been fixed.*

9. **In Discussion, lines 257-259, ‘Studies show that for samples with negative smear microscopy, the sensitivity of the TRM-TB for a sputum sample is 72.5% and for three samples it reaches almost 91%[2]’
   Please rewrite this sentence.**

*Thank you for your comment. This sentence has been removed from the discussion and a paragraph has been inserted that further details this information to facilitate understanding.*

10. **What is SUS? In line 268 same page. I believe,it is city. Then write accordingly.**

*Thank you for your comment. The acronym SUS stands for Sistema Único de Saúde and refers to the brazilian health system, which had been explained briefly in the introduction. Understanding that it can cause confusion among readers, the acronym SUS was replaced in the entire manuscript by the brazilian health system.*

11. **Across the world, sputum smear microscopy and culture are commonly used methods for the detection of pulmonary TB. For the detection of extrapulmonary TB, these methods are not good. In fact, TB diagnosis also relies on other diagnostic modalities, like hisopathology, imaging, IGRAs including molecular tests, like LAMP test, PCR, multiplex PCR, real-time PCR, etc.**

*Thank you for your comment. Three paragraphs were inserted in the discussion of studies proving the effectiveness of the rapid molecular test for tuberculosis in extrapulmonary samples. Although there are other diagnostic techniques as you mentioned, GeneXpert^®^ MTB / RIF is a reliable option for this type of sample.*

12. **In Discussion, lines 274, ‘A culture test can detect between 70% and 90% of cases; however, it requires at least 14 to 60 days to obtain a diagnosis’ is not written accurate. In fact, the sensitivity of culture in extrapulonary paucibacillary TB specimens varies from 0-80%.**

*Thank you for your comment. This information regarding the sensitivity of the culture was based on the references cited in this paragraph. In addition, I did a search and in addition to those mentioned, other articles and government documents (from Brazil and the world) reinforce this information that the sensitivity of the culture can vary between 70 and 90% when the sample was correctly collected, stored and the technique the exam was correctly performed. I think this may have caused some confusion. The sentence has been reformulated to improve understanding.*

13. **In Discussion, line 297, what do you mean by sensitivity test (ST)? Is it drug susceptibility test (DST)?**

*Thank you for your comment. Yes, the sensitivity test is the* *drug susceptibility test. The term was changed in the manuscript to facilitate understanding.*

14. **You need to compare Xpert assay at the Lab level with other routine tests for TB diagnosis like imaging, histopathology, cytology, PCR, real-time PCR, etc.Pl mention these tests in the Introduction as well as Discussion.**

*Thank you for your comment, this information was included in the introduction and discussion*

**Reviewer #2:**

**The manuscript titled as "Effectiveness and trend forecasting of tuberculosis diagnosis after the introduction of GeneXpert in a city in south-estern Brazil" is an interesting topic, and supplied the diagnosis for the care of a person with tuerculosis. The manuscript has reached the publication level of PLoS One, I recommend to accept it. I have no comments to the authors.**

*I would like to thank you for having read and evaluated the manuscript and it was very gratifying for us to know that, in your opinion, it is at the level of publication in such a prestigious journal.*
