# Supplementary material for: Effectiveness and trend forecasting of tuberculosis diagnosis after the introduction of GeneXpert in a city in south-eastern Brazil
Source: PLoS One. 2021 May 28;16(5):e0252375. doi: 10.1371/journal.pone.0252375 (PMC8162696; doi:10.1371/journal.pone.0252375)
Supplement: Attachment — Submitted filename: Response to Reviewers.docx [file pone.0252375.s004.docx]

**Dear Editor,**

Many thanks for your reply and your reviewers' comments about our manuscript **“PONE-D-20-34732 Effectiveness and trend forecasting of tuberculosis diagnosis after the introduction of GeneXpert® in a city in south-eastern Brazil”.**

The comments were appropriate to qualify/improve the manuscript. We have forwarded a letter with the changes made in the manuscript, presenting our response for each comment from reviewers, as requested. Many thanks for the comments.

Kind regards,

Berra et al.

**Reviewers' comments:**

**Reviewer #1:**

1. **The word ‘rates of disease’ in Abstract and otherwise need to be changed, e.g. ‘outcome of disease’. At few instances, rate of disease is OK but at few instances, it reads really odd.**

**Response:** *Thank you for your comment. In this case of the objective of the study, what we did from the temporal modeling was to make a prediction of the incidence rates of tuberculosis, therefore, we believe that substituting for "outcome of disease" will not keep the same meaning of the phrase, since in the present study we do not assess the outcome of tuberculosis, but work with the rates.*

**2. Tuberculosis need to be abbreviated as TB first time and later, TB can be written in Abstract as these two terms are written haphazardly.**

**Response:** *Thank you for your comment. As suggested, we introduced the word tuberculosis in full the first time it appears and replaced it with its acronym (TB) throughout the Abstract.*

**3. Lines, 261-68 of Discussion need to be rewritten. Similarly, line 269 needs attention.**

**Response:** *Thank you for your comment. Paragraphs have been modified to better understand their content.*

**4. ‘incidence rates (lines 273-274 of Discussion) for extrapulmonary TB and TB-HIV co-infection changed in structure after 2014’,the word ‘structure’ needs to be replaced. Similarly, sentence in lines 275-276 should be rewritten. It should be ‘might’ instead of ‘may’ in line 278.**

**Response:** *Thank you for your comment. The paragraph was rewritten but we chose not to remove the word "structure" since it is related to the method used and, in this way, we consider that it facilitates the reader to relate the result presented with the method used.*

**5. Line 280, in Discussion, appropriate reference shall be given. While interpreting pulmonary and extrapulmonary TB samples by molecular test (GeneXpert), sensitivity and specificity may be almost similar in the given reference, but mostly pulmonary TB exhibited a much higher sensitivity than exptrapulmonary TB. Pl quote those references and give valid reasons for that, e.g. due to paucibacillary nature of specimens, etc.**

**Response:** *Thank you for your comment. I apologize but I am not sure if I understood correctly what was suggested, but we have added two paragraphs to the discussion on this issue of extrapulmonary samples.*

**6. Lines, 284-90, shall be rewritten. What do you mean positive in people who actually have TB? I must point out that GeneXpert mostly does not perform well with Pleural TB cases as it reveals poor sensitivity (Pl see Review of i) Denkinger CM, Schumacher SG, Boehme CC, Dendukuri N, Pai M, Steingart KR. Xpert MTB/RIF assay for the diagnosis of extrapulmonary tuberculosis: a systematic review and meta-analysis. Eur. Respir. J. 44, 435–446 (2014). ii) Sehgal IS, Dhooria S, Aggarwal AN, Behera D, Agarwal R. Diagnostic performance of Xpert MTB/RIF in tuberculous pleural effusion: systematic review and meta-analysis. J. Clin. Microbiol. 54(4), 1133–1136 (2016).**

**Response:** *Thank you for your comment. We rewrote the sentence in order to make it clear that we are talking about false positive and negative results and added a note about the lower effectiveness of the test for pleural samples. Thanks for sending the references, they were added and cited in the text.*

**7. Lines 297-301, also, write that culture is labor-intensive method and requires skillful technicians. You are probably mentioning culture on LJ medium. What about culture on MGIT-960 system? Write plus and negative points for that.**

**Response:** *Thank you for your comment. We inserted a paragraph in the discussion comparing the results of the culture carried out in solid medium and automated liquid medium.*

**8. Line 306 of Discussion, what do you mean by ‘consequent change in structure in the time series’? Please write clearly. Similarly, lines 322-323 need attention.**

**Response:** *Thanks for your comment, the phrases have been revised and rewritten to make your content clearer.*

**9. Line 326, further studies in which direction will improve the understanding of this test? There is probably no need to further understand the test. Yes, we can probably improve the sensitivity by using Xpert Ultra. Also, may be the technology could be improved as in case of Xpert Omni. You need to mention these points.**

**Response:** *Thank you for your comment. We added a paragraph about GeneXpert Ultra and Omni.*

**10. Line 328, ‘studies of this nature’ need to be replaced. Similarly, lines 335-338 need to be rewritten.**

**Response:** *Thank you for your comment. We changed the word "nature" to "design" and the last paragraph of the conclusion has been rewritten, as suggested.*
