# Supplementary material for: Effectiveness and trend forecasting of tuberculosis diagnosis after the introduction of GeneXpert in a city in south-eastern Brazil
Source: PLoS One. 2021 May 28;16(5):e0252375. doi: 10.1371/journal.pone.0252375 (PMC8162696; doi:10.1371/journal.pone.0252375)
Supplement: Attachment — Submitted filename: Response to Reviewers.docx [file pone.0252375.s005.docx]

**Dear Editor,**

Many thanks for your reply and your reviewers' comments about our manuscript **“PONE-D-20-34732 Effectiveness and trend forecasting of tuberculosis diagnosis after the introduction of GeneXpert® in a city in south-eastern Brazil”.**

The comments were appropriate to qualify/improve the manuscript. We have forwarded a letter with the changes made in the manuscript, presenting our response for each comment from reviewers, as requested. Many thanks for the comments.

Kind regards,

Berra et al.

**Editors’ comments:**

1. **Please review your reference list to ensure that it is complete and correct. If you have cited papers that have been retracted, please include the rationale for doing so in the manuscript text, or remove these references and replace them with relevant current references. Any changes to the reference list should be mentioned in the rebuttal letter that accompanies your revised manuscript. If you need to cite a retracted article, indicate the article’s retracted status in the References list and also include a citation and full reference for the retraction notice.**

**Response:** *Thank you for your comment. All references have been revised.*

**Reviewers' comments:**

**Reviewer #1:**

**Manuscript is improved but still there are pitfalls. English needs to be improved tremendously so that it reads well.**

**Response:** *Thank you for reevaluating the manuscript once again. We inform that the manuscript had been revised in English by a specialized company (https://www.proof-reading-service.com) by natives. Again we send the manuscript to have its writing corrected, we hope it has improved.*

1. **Conclusions section of Abstract need to be rewritten.**

**Response:** *The conclusion of Abstract has been modified.*

2. **HIV on lines 51 and 53. First time you wrote HIV, second time you write human immunodeficiency virus (HIV). Not correct. In fact, writing HIV itself is fine at both the places as this term so common. Similarly, when you write first time, Mycobacterium tuberculosis is fine, but later when you write M. tuberculosis is fine.**

**Response:** *In fact, the first time that the acronym HIV appears is in the first paragraph of the introduction, along with the acronym AIDS, in which both acronyms were written in full.*

*In the lines in which you cited, the second appearance was spelled out in full because it is a new acronym, TB-HIV co-infection, therefore, just being the union of two acronyms that have already appeared in the text, we write its meaning in full in order to follow the journal's norms.*

*Regarding* Mycobacterium tuberculosis*, we leave it in full the first time it appears in the text and at other times we change it to* M. tuberculosis*, as recommended.*

3. **Line 60, it should be ‘****prolonged turnaround time’.**

**Response:** *Thanks, this has been fixed.*

4. **Line 74, it is highly sensitive for pulmonary TB, but not for extrapulmonary TB.**

**Response:** *Thanks, this has been fixed.*

5. **Lines 282-86, need to write clearly that when culture was employed as the gold standard, then the sensitivity and specificity for GeneXpert were determined for pulmonary and extrapulmonary TB. Please read this Ref 23 carefully and then write.**

**Response:** *Thank you. that information was inserted in the same paragraph.*

6. **Lines 288-89, need to specifically mention here GeneXpert assay and not molecular tests because there are many other molecular tests. Moreover, at times with many extrapulmonary TB samples, GeneXpert shows poor sensitivity so ‘good test performance’ shall be replaced.**

**Response***: Thank you, the paragraph has been rewritten.*

7. **Lines 294-95 shall be rewritten.**

**Response***: Thank you, the paragraph has been rewritten.*

8. **Line 300, it shall be written as ‘not good sensitivity with Xpert assay’ instead of efficacy with molecular test. This is because there are many other molecular tests.**

**Response:** *Thanks, this has been fixed.*

9. **Line 311 ‘performing the exam’ is not appropriate. Pl re-write. In fact lines 311-13 need to be rewritten.**

**Response***: Thank you, the paragraph has been rewritten.*

10. **Line please elaborate about liquid culture e.g. MGIT-960 and its advantages over sold media.**

**Response***: Three paragraphs were added to the discussion session, better addressing the culture in liquid medium and also comparing it with solid medium L-J.*

11. **Lines 330-331 still not clear, please rewrite.**

**Response***: The sentence was rewritten again.*

12. **Line 374, what are those certain ways?**

**Response:** *Thanks, this has been fixed.*

13. **Lines 374-76, need to be rewritten.**

**Response***: Thank you, the paragraph has been rewritten.*
