# Supplementary material for: Effectiveness and trend forecasting of tuberculosis diagnosis after the introduction of GeneXpert in a city in south-eastern Brazil
Source: PLoS One. 2021 May 28;16(5):e0252375. doi: 10.1371/journal.pone.0252375 (PMC8162696; doi:10.1371/journal.pone.0252375)
Supplement: Attachment — Submitted filename: Comments-18-4-21.docx [file pone.0252375.s006.docx]

**Comments**

1. Conclusion of Abstract, line 45, it shall be ‘it is possible’, instead of ‘is it possible’.

2. In Intro, line 53, overcoming AIDS today, you need to reframe this sentence.

3. Line 63, shall be ‘considered as the gold standard’.

4. Line 72, it shall be based on the real-time PCR, instead of ‘polymerase chain reaction (PCR), also known as real-time PCR’. This is because it is incorrect to write that PCR is also known as real-time PCR, two techniques are different.

5. Line 80, GeneXpert, in general, is not considered to be a cost-effective test especially in developing countries. Rather, it is considered as a major drawback of this technique as it is not cost-effective.

6. Lines, 77 and 84, as well as 259, it shall be ‘for the detection of’ (and not ‘in the detection of ’).

7. Line 270, it shall be ‘decreases to 57 - 78.2%’ instead of drops to 57 to 78.2%.

8. Lines, 270-274 shall be split into two sentences and written with clarity.

9. Lines 296 and 298 as well as line 300, it shall be sputum samples and not lung samples.

10. Lines 301-303 are incorrect. ‘it is important that further studies are carried out with this type of material as this result may change according to the number of samples tested’, need to be re-written. It is not only the sample size, but different nature of extrapulmonary TB specimens and their collection across diverse geographical locations of entire world.

11. Lines 305 and 311 are repeated (‘Thus, the performance of the GeneXpert® system in extrapulmonary samples is still not entirely clear’).

12. Line 311, ‘again we should stress’, it shall be rewritten.

13. Line 316, ‘to confirm your diagnosis’, is wrong. Please omit the word ‘your’ here.

14. Lines 327, sputum smear and culture examinations are for pulmonary TB, only. You need to mention. That. For extrapulmonary TB, sputum is not taken; rather other bodily fluids undergo smear / culture examination.

15. Lines 354 and 355, two times high cost. Please rewrite.
