# Supplementary material for: Effectiveness and trend forecasting of tuberculosis diagnosis after the introduction of GeneXpert in a city in south-eastern Brazil
Source: PLoS One. 2021 May 28;16(5):e0252375. doi: 10.1371/journal.pone.0252375 (PMC8162696; doi:10.1371/journal.pone.0252375)
Supplement: Attachment — Submitted filename: Response to Reviewers.docx [file pone.0252375.s007.docx]

**Dear Editor,**

Many thanks for your reply and your reviewers' comments about our manuscript **“PONE-D-20-34732 Effectiveness and trend forecasting of tuberculosis diagnosis after the introduction of GeneXpert® in a city in south-eastern Brazil”.**

The comments were appropriate to qualify/improve the manuscript. We have forwarded a letter with the changes made in the manuscript, presenting our response for each comment from reviewers, as requested. Many thanks for the comments.

Kind regards,

Berra et al.

**Editors’ comments:**

1. **Please review your reference list to ensure that it is complete and correct. If you have cited papers that have been retracted, please include the rationale for doing so in the manuscript text, or remove these references and replace them with relevant current references. Any changes to the reference list should be mentioned in the rebuttal letter that accompanies your revised manuscript. If you need to cite a retracted article, indicate the article’s retracted status in the References list and also include a citation and full reference for the retraction notice.**

**Response:** *Thank you for your comment. All references have been revised.*

**Reviewers' comments:**

**Reviewer #1:**

1. **Conclusion of Abstract, line 45, it shall be ‘it is possible’, instead of ‘is it possible’.**

**Response:** *Thank you, this has been fixed.*

**2. In Intro, line 53, overcoming AIDS today, you need to reframe this sentence.**

**Response:** *Thanks for your comment, this sentence has been modified to improve your understanding.*

**3. Line 63, shall be ‘considered as the gold standard’.**

**Response:** *Thank you, this has been fixed.*

**4. Line 72, it shall be based on the real-time PCR, instead of ‘polymerase chain reaction (PCR), also known as real-time PCR’. This is because it is incorrect to write that PCR is also known as real-time PCR, two techniques are different.**

**Response:** *Thank you, this has been fixed.*

**5. Line 80, GeneXpert, in general, is not considered to be a cost-effective test especially in developing countries. Rather, it is considered as a major drawback of this technique as it is not cost-effective.**

**Response:** *Thank you for your comment, we put this observation at the end of that same indicated paragraph.*

**6. Lines, 77 and 84, as well as 259, it shall be ‘for the detection of’ (and not ‘in the detection of ’).**

**Response:** *Thank you, this has been fixed.*

**7. Line 270, it shall be ‘decreases to 57 - 78.2%’ instead of drops to 57 to 78.2%.**

**Response:** *Thank you, this has been fixed.*

**8. Lines, 270-274 shall be split into two sentences and written with clarity.**

**Response:** *Thanks for your comment, the paragraph has been rewritten.*

**9. Lines 296 and 298 as well as line 300, it shall be sputum samples and not lung samples.**

**Response:** *Thank you, this has been fixed.*

**10. Lines 301-303 are incorrect. ‘it is important that further studies are carried out with this type of material as this result may change according to the number of samples tested’, need to be re-written. It is not only the sample size, but different nature of extrapulmonary TB specimens and their collection across diverse geographical locations of entire world.**

**Response:** *Thank you for your comment. These observations have been inserted in the paragraph.*

**11. Lines 305 and 311 are repeated (‘Thus, the performance of the GeneXpert® system in extrapulmonary samples is still not entirely clear’).**

**Response:** *Thank you, this has been fixed.*

**12. Line 311, ‘again we should stress’, it shall be rewritten.**

**Response:** *Thank you, this has been fixed.*

**13. Line 316, ‘to confirm your diagnosis’, is wrong. Please omit the word ‘your’ here.**

**Response:** *Thank you, this has been fixed.*

**14. Lines 327, sputum smear and culture examinations are for pulmonary TB, only. You need to mention. That. For extrapulmonary TB, sputum is not taken; rather other bodily fluids undergo smear / culture examination.**

**Response:** *Thank you for your comment. This observation was inserted in the paragraph in question.*

**15. Lines 354 and 355, two times high cost. Please rewrite.**

**Response:** *Thank you, this has been fixed.*
