# Supplementary material for: Effectiveness and trend forecasting of tuberculosis diagnosis after the introduction of GeneXpert in a city in south-eastern Brazil
Source: PLoS One. 2021 May 28;16(5):e0252375. doi: 10.1371/journal.pone.0252375 (PMC8162696; doi:10.1371/journal.pone.0252375)
Supplement: Attachment — Submitted filename: Comments-5-5-21.docx [file pone.0252375.s008.docx]

**Comments**

1. Lines 299-302 and lines 303-312 are repetition; therefore lines 299-302 shall be omitted in discussion.

Moreover, lines 303-312 need to be rewritten, as English is of substandard.

2. Line 351: the term MGIT-960 System shall be written above along with automated liquid culture system in lines 344-345.
